# Enhanced chlorhexidine skin penetration with 1,8-cineole

**DOI:** 10.1186/s12879-017-2451-4

**Published:** 2017-05-17

**Authors:** A. L. Casey, T. J. Karpanen, B. R. Conway, T. Worthington, P. Nightingale, R. Waters, T. S. J. Elliott

**Affiliations:** 1 Department of Clinical Microbiology, University Hospitals Birmingham NHS Foundation Trust, Edgbaston, B15 2WB Birmingham, UK; 2 Department of Pharmacy, University of Huddersfield, Queensgate, Huddersfield, HD1 3DH UK; 3 School of Life and Health Sciences, Aston University, B4 7ET Birmingham, UK; 4 Wolfson Computer Laboratory, University Hospitals Birmingham NHS Foundation Trust, B15 2TH Birmingham, UK; 5 Department of Burns and Plastics, University Hospitals Birmingham NHS Foundation Trust, B15 2WB Birmingham, UK; 6 Clinical Governance, The Queen Elizabeth Hospital Birmingham, University Hospitals Birmingham NHS Foundation Trust, Edgbaston, B15 2TH Birmingham, UK

**Keywords:** Skin antisepsis, Antiseptic penetration, Terpene

## Abstract

**Background:**

Chlorhexidine (CHG) penetrates poorly into skin. The purpose of this study was to compare the depth of CHG skin permeation from solutions containing either 2% (*w*/*v*) CHG and 70% (*v*/v) isopropyl alcohol (IPA) or 2% (*w*/*v*) CHG, 70% (*v*/v) IPA and 2% (*v*/v) 1,8-cineole.

**Methods:**

An ex-vivo study using Franz diffusion cells was carried out. Full thickness human skin was mounted onto the cells and a CHG solution, with or without 2% (*v*/v) 1,8-cineole was applied to the skin surface. After twenty-four hours the skin was sectioned horizontally in 100 μm slices to a depth of 2000 μm and the concentration of CHG in each section quantified using high performance liquid chromatography (HPLC). The data were analysed with repeated measures analysis of variance.

**Results:**

The concentration of CHG in the skin on average was significantly higher (33.3% [95%, CI 1.5% - 74.9%]) when a CHG solution which contained 1,8-cineole was applied to the skin compared to a CHG solution which did not contain this terpene (*P* = 0.042).

**Conclusions:**

Enhanced delivery of CHG can be achieved in the presence of 1,8-cineole, which is the major component of eucalyptus oil. This may reduce the numbers of microorganisms located in the deeper layers of the skin which potentially could decrease the risk of surgical site infection.

## Background

Most pathogens that cause surgical site infection are endogenous, primarily from the patient’s skin [1]. Chlorhexidine (CHG) is often recommended for skin antisepsis prior to invasive procedures [2], http://hcai.dh.gov.uk/files/2011/03/2011-03-14-HII-Prevent-Surgical-Site-infection-FINAL.pdf however it penetrates poorly into skin [3, 4]. This may reflect binding of antiseptic compounds including CHG to intercellular lipids in the stratum corneum [5]. Endogenous microorganisms may therefore persist in the deeper layers of the skin despite the application of topical antiseptics such as CHG. It is therefore important to determine methods for increasing the penetration of CHG into human skin to potentially facilitate eradication of these microorganisms during application of antiseptics and subsequently reduce the risk of infection.

Eucalyptus oil has been successfully used as a skin penetration enhancer to deliver drugs such as steroid hormones [6]. Interestingly, this oil also enhances the delivery of CHG into the epidermis and dermis [7]. In addition eucalyptus oil exhibits antimicrobial activity and in combination with CHG acts synergistically [8, 9]. Whilst eucalyptus oil has exhibited a more potent antimicrobial activity than 1,8 cineole in suspension tests, 1,8 cineole combined with CHG demonstrates a synergistic effect in both suspension and biofilm tests [10]. Since the composition of crude eucalyptus oil can vary, a purified solution of the main constituent terpene, 1,8 cineole may offer a potential defined alternative to both enhance CHG intradermal delivery as well as enhance antimicrobial activity. This is also supported by the previous observations that 1,8 cineole is a highly effective penetration enhancer for both 5-fluorouracil and estradiol [11]. The mechanism by which terpenes enhance skin permeation is thought to be by interaction with lipids present in the intercellular region of the stratum corneum [12, 13].

Since 1,8-cineole enhances skin permeation of some drugs and is the main constituent of eucalyptus oil, which enhances CHG penetration, we hypothesised that 1,8-cineole will enhance the skin penetration of CHG. The objective of this present study was therefore to quantify CHG penetration into human skin following application of 2% (*w*/*v*) CHG and 70% (*v*/v) isopropyl alcohol (IPA) with or without the addition of 2% (*v*/v) 1,8-cineole. To our knowledge, this is the first evaluation of potential enhancement of skin penetration of CHG using 1,8-cineole.

## Methods

### Donor skin

Following donor consent, full thickness human skin was obtained from three women aged 51, 52, and 53 years. All skin was excess abdominal tissue following deep inferior epigastric perforators (DIEP) flap surgery for breast reconstruction and was frozen at −20 °C on the day of excision and used within four weeks. Ethics committee approval was granted by NRES Committee West Midlands, UK (REC: 14/WS/1012). All skin donors consented for their tissue to be used in research.

### Skin penetration of CHG using an ex -vivo skin model

Studies were performed as in previous ex vivo skin penetration investigations undertaken by our group [3, 7]. In brief, the receptor compartments of Franz diffusion cells were filled with 15 mL of sterile PBS and maintained at 37 °C. Eighteen pieces of full thickness skin (six pieces from each of three donors) measuring 3 cm × 3 cm were thawed before mounting onto the cells with the stratum corneum facing upwards. Any entrapped air between the skin and the receptor fluid was removed. The skin surface was blotted dry and left to equilibrate for 30 min. Two of the skin samples from each donor had 100 μL of a solution containing 2% (*w*/*v*) CHG (Sigma Aldrich, Poole, UK) and 70% (*v*/v) IPA (Fisher Scientific, Loughborough, UK) applied to the surface and two skin samples from each donor had 100 μL of a solution containing 2% (*w*/*v*) CHG, 70% (*v*/v) IPA and 2% (*v*/v) 1,8-cineole (Sigma Aldrich, Poole, UK) applied. The solutions were spread evenly across the skin surface (3.14 cm^2^) and left at room temperature for 24 h. The two remaining skin samples from each donor were mounted onto the Franz cells without the application of antiseptic. These were used to quantify background CHG levels present in the skin as all donor patients received pre-operative skin preparation with 2% (*w*/*v*) CHG in 70% (*v*/v) IPA [ChloraPrep® CareFusion UK Ltd., Basingstoke, UK] prior to skin excision.

The skin samples were frozen with a cryospray (Leica, Milton Keynes, UK) and three punch biopsies (7 mm in diameter, totalling an area of 1.15 cm^2^) removed from each sample. This equated to approximately 732.5 μg of CHG applied to each punch biopsy area. In comparison, when ChloraPrep® one-step applicators are used in clinical situations, according to the manufacturers’ instructions, a minimum of 22.98 μL 2% (*w*/*v*) antiseptic solution (equal to 459.6 μg CHG) per cm^2^ of skin is delivered. The solution used in our study therefore provided a comparable level of CHG to that by ChloraPrep® in the clinical scenario. Skin biopsies were subsequently sectioned horizontally with a cryotome (Leica, Milton Keynes, UK) into 100 μm sections (to a depth of 2000 μm to encompass some depth of follicular sectioning). Corresponding sections from the three biopsies were pooled in separate sterile polypropylene centrifuge tubes (Fisher Scientific, Loughborough, UK) and the weight of each sample determined.

### Quantification of CHG

Quantification of CHG was determined using a validated high performance liquid chromatography (HPLC) method as described previously [3, 7]. Extraction of CHG from skin sections was carried out by the addition of 1 mL of HPLC isocratic mobile phase [75% (*v*/v) HPLC grade methanol (Fisher Scientific), 25% distilled water, 0.1% (*v*/v) HPLC grade diethylamine (Sigma Aldrich), and 0.005 M sodium heptane sulphonate (Sigma Aldrich) adjusted to pH 4 with HPLC grade glacial acetic acid (Fisher Scientific)] to each centrifuge tube containing corresponding sections from three biopsies which were incubated at 37 °C for one hour. All samples were then vortex-mixed for 30 s, centrifuged for 10 min at 6000 g, filtered through a 0.45 μm filter, and the CHG quantified with an Agilent 1200 series HPLC system (Agilent Technologies, Stockport, UK). Samples were run at room temperature at 1.2 mL/min through a reverse-phase chromatography column [CPS-2 Hypersil 5-μm column; 150 mm × 4.6 mm (Fisher Scientific)] with UV detection at 254 nm. CHG extraction was validated prior to the study and HPLC with every run [3, 7].

### Statistical analysis

To improve the validity of assumptions of normal distributions, the 2% (*w*/*v*) CHG and 70% (*v*/v) IPA, 2% (*w*/*v*) CHG and 70% (*v*/v) IPA with 2% (*v*/v) 1,8-cineole and background values at each depth were log transformed. These values were used in the statistical analysis. The data were analysed with repeated measures analysis of variance with two within subjects factors, a) background, with and without 1,8-cineole and b) depth below the skin surface. The Greenhouse-Geisser test was used to assess the significance of F values. Repeated contrasts were used to compare concentrations at successive depths. SPSS version 22 (IBM, Armonk, NY) was used for data analysis.

## Results

### Background levels of CHG in full thickness skin

**Fig. 1:**
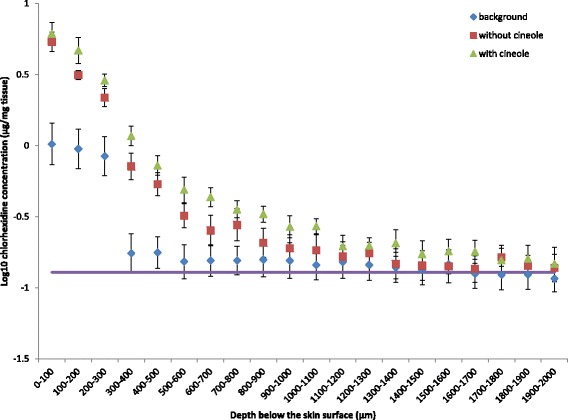
Concentration of CHG in skin following exposure to 2% CHG/70% IPA with or without 2% (*v*/v) 1,8-cineole. Penetration profile showing the concentration of CHG in excised human skin (0-2000 μm depth) after exposure to 2% (*w*/*v*) CHG in 70% (*v*/v) IPA with and without 2% (*v*/v) 1,8-cineole (mean ± SEM). Background levels of CHG derived from preoperative skin antisepsis, without any further CHG added are also presented. The purple line represents the MIC value of CHG against MRSA (N315) in a biofilm [9]. The assumption that 1 g of skin is equal to 1 mL was made. The concentration of CHG in the skin was significantly higher in the CHG/IPA with 1,8-cineole group compared to the CHG/IPA alone (*P* = 0.042). The size of the effect did not vary according to the depth of penetration into the skin (*P* = 0.311), indicating that the concentration of CHG was higher at all depths with the addition of 1,8-cineole

All the donor patients had CHG applied preoperatively using ChloraPrep® according to manufacturer’s instructions. For samples which were not treated with any further antiseptic material, CHG was detected in all layers of the sectioned skin (Fig. 1). There was a significant fall in CHG concentration between the 200–300 μm and 300–400 μm measurements (0.667 μg/mg tissue, *P* < 0.001).

### Levels of CHG in full thickness skin following application of 2% (*w*/*v*) CHG and 70% (*v*/v) IPA or 2% (*w*/*v*) CHG, 70% (*v*/v) IPA, and 2% (*v*/v) 1,8-cineole

The concentration of CHG recovered from the skin at various depths is shown in Fig. 1. In the CHG/IPA alone group, there was a significant decrease in the CHG concentration with every 100 μm section skin between 100 μm and 700 μm below the skin surface (*P* = 0.028, 0.012, <0.001, 0.009, 0.008 and 0.026 respectively). For the CHG/IPA with 1,8-cineole group, CHG levels decreased for every 100 μm depth of skin between 200 μm and 600 μm below the skin surface (*P* = 0.026, 0.002, 0.010 and 0.041 respectively). The concentration of CHG in the skin was on average significantly higher in both the CHG/IPA and CHG/IPA with 1,8-cineole groups compared to background levels (*P* = 0.015 and *P* = 0.020, respectively). The size of the effect varied according to the depth of penetration into the skin (*P* = 0.004 and *P* = 0.011, respectively). The difference between background and treatment values tended to decrease with increasing depths. The concentration of CHG in the skin was on average significantly higher in the CHG/IPA with 1,8-cineole group compared to the CHG/IPA alone (*P* = 0.042). However the size of the effect did not vary according to the depth of penetration into the skin (*P* = 0.311), with there being no evidence that the true effect varied with depth. The concentrations of CHG in the skin following application of the CHG solution containing 1,8-cineole were on average 33.3% higher (95%, CI 1.5% - 74.9%) than when the CHG solution without 1,8-cineole was applied.

### Detection of CHG from the total applied to the skin surface

A median of 40.46% (range = 9.99–61.89%) of the total CHG applied to the skin surface was detected.

## Discussion

In this study low levels of CHG were recovered from skin to which no additional antiseptic was applied during experimentation. This CHG originated from the 2% (*w*/*v*) CHG in 70% (*v*/v) IPA pre-operative skin preparation which all donor patients received following manufacturer’s guidelines, immediately prior to their surgery. The use of this antiseptic follows recommendations for preoperative skin preparations [2] http://hcai.dh.gov.uk/files/2011/03/2011-03-14-HII-Prevent-Surgical-Site-infection-FINAL.pdf. Chlorhexidine penetrates skin poorly which was exemplified in this present study by the significant decrease in CHG concentration between the 200-300 μm and 300-400 μm sections. This may in part be due to the large molecular size of CHG or its binding to skin lipids [4]. In addition, the combination of CHG and IPA has been reported to reduce the initial skin penetration of CHG even further after a two minute (but not a 30 min) application to the skin surface and this may also offer an explanation for the relatively low levels of CHG detected in the donor skin [4]. Conversely, a synergistic effect between 1,8-cineole and ethanol on the percutaneous absorption of diclofenac sodium has been observed [14]. Furthermore, it has been demonstrated that the combination of eucalyptus oil, CHG and IPA in a hard surface wipe reduced the time required for removal of biofilms compared to CHG and IPA alone [13]. This suggests that the addition of eucalyptus oil or 1,8-cineole may reduce the risk of any adverse effects on penetration that IPA may induce.

In this study IPA- and aqueous CHG solutions were not directly compared, however it was demonstrated that skin penetration of CHG from the recommended 2% (*w*/*v*) CHG in 70% (*v*/v) IPA solution was significantly enhanced with the addition of 2% (*v*/v) 1,8-cineole. In addition the effect on CHG penetration by evaporation of the IPA or loss from the skin surface by vaporisation of some of the cineole was not determined as we wished to emulate the clinical scenario of applying skin antisepsis. It is thought that terpenes enhance lipophilic drug penetration by increasing the partition coefficient (partitioning of drug between vehicle and stratum corneum), as well as hydrophilic drug penetration by increasing the diffusion coefficient [15]. Partitioning 1,8-cineole in the skin lipids is heterogeneous, leading to both ordered and disordered areas in stratum corneum lipids [11].

Although in vitro MIC levels of CHG were detected down to 2000 μm below the skin surface in the presence or absence of 1,8-cineole, it is unknown how the MIC detected using in vitro methods relates to the MIC in skin itself [9]. It would seem likely that antimicrobial activity may be reduced in the skin with protein binding and bacteria being present in biofilms, however it is currently unknown how 1,8 cineole influences CHG binding and inactivation [9]. Indeed, although we have demonstrated increased skin permeation of CHG in the presence of 1,8- cineole, further studies are required to investigate if this is translated into enhanced antimicrobial activity (both from the CHG and synergistic combination of CHG and 1,8 cineole) [9].

## Conclusions

Since the composition of crude eucalyptus oil can vary according to source, 1,8 cineole may therefore offer a defined alternative which could be used in a licensed skin antiseptic.

The higher concentrations of CHG detected in the donor skin may improve the antimicrobial activity of preoperative skin preparations thereby reducing the risk of infection associated with procedures involving skin incision Howeverfurther studies are required to investigate if the observed enhanced CHG skin penetration results in improved antimicrobial activity in situ.

## Abbreviations

CHG: Chlorhexidine
DIEP: Deep inferior epigastric perforators
HPLC: High performance liquid chromatography
IPA: Isopropyl alcohol
MIC: Minimum inhibitory concentration
PBS: Phosphate buffered saline.
